# A First-Principles Calculation Study of the Catalytic Properties of Two-Dimensional Bismuthene Materials for Carbon Dioxide Reduction

**DOI:** 10.3390/ma18030594

**Published:** 2025-01-28

**Authors:** Chang-Tian Wang, Qinchi Yue, Changhao Wang, Yuanji Xu, Chang Zhou

**Affiliations:** 1 Beijing Advanced Innovation Center for Materials Genome Engineering, Department of Physical Chemistry, University of Science and Technology Beijing, Beijing 100083, China; ctwang@ustb.edu.cn; 2 Key Laboratory of Advanced Functional Materials of Education Ministry of China, College of Materials Science and Engineering, Beijing University of Technology, Beijing 100124, China; yueqinchiy@163.com (Q.Y.); wangch33@bjut.edu.cn (C.W.); 3 Institute for Applied Physics, University of Science and Technology Beijing, Beijing 100083, China; yuanjixu@ustb.edu.cn; 4 State Key Laboratory for Advanced Metals and Materials, University of Science and Technology Beijing, Beijing 100083, China

**Keywords:** bismuthene, 2D materials, first-principles calculations, carbon dioxide (CO_2_), catalyze

## Abstract

The electrochemical reduction of carbon dioxide (${\mathrm{CO}}_{2}$) at room temperature into industrial chemicals and energy products offers a promising strategy to mitigate atmospheric greenhouse gas emissions. In this study, bismuthene was employed as a catalyst for ${\mathrm{CO}}_{2}$ reduction reaction (${\mathrm{CO}}_{2}$RR). Through first-principles calculations, we evaluated the ${\mathrm{CO}}_{2}$RR catalytic activities of bismuth (Bi) on the (001) and (012) surfaces, analyzing the mechanisms underlying these activities. Surface energy calculations for monolayer and multilayer bismuthene confirmed that monolayer bismuthene is stable and suitable for catalytic applications. Adsorption free energies of intermediates showed that formic acid is the primary product. Furthermore, it is found that the Bi(012) surface has a lower free energy barrier than Bi(001) in the ${\mathrm{CO}}_{2}$RR process, representing the higher catalytic activity. These results provide theoretical insights for designing bismuthene-based ${\mathrm{CO}}_{2}$RR catalysts with reduced overpotential, improved efficiency and enhanced selectivity, particularly enhancing catalyst selectivity.

## 1. Introduction

Since Geim et al. successfully exfoliated single-layer graphene using adhesive tape in 2004 [1], the intriguing electronic properties of graphene have ignited intense interest in layered two-dimensional (2D) materials. As research into 2D materials has advanced, researchers have discovered that these materials exhibit numerous exciting novel properties, such as larger bandgaps and tunable electronic, optoelectronic, catalytic, and photoelectrochemical properties, compared to their bulk counterparts [2,3,4,5,6,7]. Thus, 2D materials are set to be a key foundation for future advances in electronics, optoelectronics, energy conversion, and storage. Research on ${\mathrm{CO}}_{2}$ catalytic reduction supports a sustainable carbon cycle, helping to reduce the greenhouse effect and tackle the energy crisis [8].

Two-dimensional electrocatalysts enriched with nanostructures represent a common and effective approach to enhancing catalytic performance. Due to their larger specific surface area, 2D electrocatalysts, particularly those with abundant edge structures containing unsaturated sites [9], provide significantly more active catalytic sites compared to bulk materials. Bismuth (Bi), characterized by higher-order topological properties, is classified as a higher-order topological insulator (HOTI). The bulk–boundary correspondence under time-reversal symmetry protection [10] suggests that these unique physicochemical properties endow low-dimensional Bi metallic catalysts with unparalleled catalytic performance compared to their bulk counterparts. Theoretically, it is predicted that one to eight bismuth (111) bilayers form a 2D topological insulator, while films exceeding eight bismuth (111) bilayers in thickness transition into a topologically trivial phase [11]. Bismuthene is a material formed by a single layer of bismuth atoms arranged in a two-dimensional honeycomb structure.

Among the products of the ${\mathrm{CO}}_{2}$ reduction reaction (${\mathrm{CO}}_{2}$RR) are compounds such as CO, ${\mathrm{CH}}_{4}$, $C_{2}$$H_{4}$, HCOOH, and $C_{2}$$H_{5}$OH. Several electron and proton transfer processes are involved. ${\mathrm{CO}}_{2}$ can be reduced to a range of gaseous and liquid products through various pathways, including hydrocarbons (such as ${\mathrm{CH}}_{4}$ and $C_{2}$$H_{4}$), alcohols (such as ${\mathrm{CH}}_{3}$OH and $C_{2}$$H_{5}$OH), carbon monoxide (CO), and formic acid (HCOOH) [12,13,14,15]. The intermediates of multi-electron transfer reactions are numerous [16,17,18], and the reduction process is complex [19,20], leading to suboptimal utilization of electrical energy and low Faradaic efficiency [21,22]. Moreover, due to the high overpotential required for the activation of ${\mathrm{CO}}_{2}$ molecules, transferring an electron from ${\mathrm{CO}}_{2}$ requires a potential more negative than −1.90 V (relative to the standard hydrogen electrode (SHE)) [23]. Sliwa et al. developed a scalable, room-temperature method for synthesizing homogeneous AgCu alloy nanoparticles with tunable Cu content, achieving high efficiency and selectivity in ${\mathrm{CO}}_{2}$ reduction at mild overpotentials, making them highly effective for electrocatalysis [24]. Experiments conducted by Sun et al. have confirmed the topological nature of 2D bismuth (111) films on ${\mathrm{NbSe}}_{2}$ substrates [25]. Bismuthene has demonstrated excellent catalytic activity and high product selectivity in electrochemical ${\mathrm{CO}}_{2}$ reduction [26,27,28,29,30,31,32,33], making it highly promising for ${\mathrm{CO}}_{2}$ reduction reaction (${\mathrm{CO}}_{2}$RR) catalysis. For the Bi(001) surface, recent studies have reported the related experimental and theoretical work. Siinor et al. studied the application of the Bi(001) single-crystal plane electrode to impedance spectroscopy [34]. Bi(001) is also used as a substrate in grown rubrene monolayers [35].

This study employs density functional theory to investigate the potential of bismuthene as a catalyst for ${\mathrm{CO}}_{2}$ reduction. We analyzed the structural stability of bismuthene with varying numbers of layers by calculating the surface energy while considering spin–orbit coupling (SOC) [36]. The results confirmed that monolayer bismuthene has the lowest surface energy, indicating superior stability. By calculating the surface adsorption free energies of various intermediates involved in ${\mathrm{CO}}_{2}$RR on the monolayer Bi(001) and Bi(012) surfaces, we constructed potential diagrams, which demonstrated that formic acid is the catalytic product of ${\mathrm{CO}}_{2}$RR and that the Bi(012) surface exhibits higher catalytic activity compared to the Bi(001) surface. We find that this catalyst exhibits a reaction advantage for ${\mathrm{CO}}_{2}$RR over the hydrogen evolution reaction (HER). Electronic band structures reveal that different intermediates have distinct effects on the electronic properties of the systems, providing an explanation for their catalytic activity from the perspective of electronic band theory. This work provides valuable theoretical insights for designing bismuthene-based ${\mathrm{CO}}_{2}$RR catalysts with desirable properties, including lower overpotential, higher selectivity, and enhanced catalytic efficiency.

## 2. Computational Method and Materials

The first-principles calculations are based on the density functional theory (DFT) as implemented in the Vienna ab initio simulation package (VASP) [37,38]. To model the interaction between ionic cores and valence electrons, the projector-augmented-wave (PAW) method [39,40] was used, with valence electrons employed as 6$s^{2}$$p^{3}$ for Bi, 2$s^{2}$$p^{2}$ for C, 2$s^{2}$$p^{4}$ for O, and 1$s^{1}$ for H. The exchange-correlation interactions are considered in the generalized gradient approximation (GGA) using the Perdew–Burke–Ernzerhof (PBE) functional [41,42]. A plane-wave basis set with an energy cutoff of 550 eV is used. The calculations were also performed with the DFT-D3 methods developed by Grimme et al., which consider the dispersion correction and adds the Van der Waals interaction to the conventional Kohn–Sham DFT potential energy [43,44]. The spin–orbit coupling (SOC) effect is considered in calculations [36].

**Figure 1:**
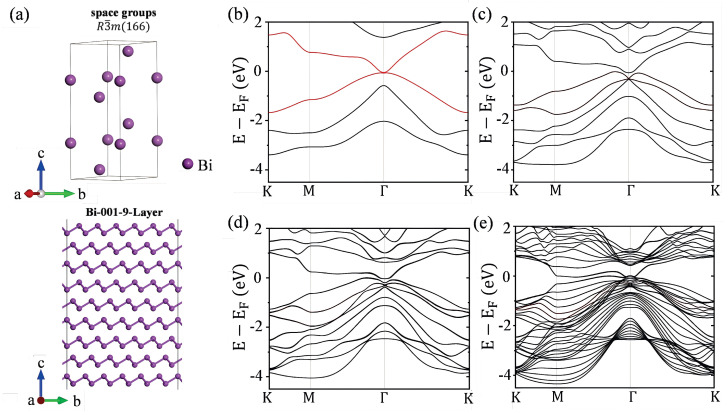
(**a**) The upper part illustrates the primitive cell of the bulk Bi structure, while the lower part shows the Bi-001-9-Layer. The band structures of the Bi-001-i-Layer without SOC are presented in (**b**–**e**) for i = 1, 2, 3, 9, respectively. The red lines in (**b**) represent the surface states of the 1-layer Bi(001) surface.

The bulk Bi structure is shown in the upper part of Figure 1a, which has a 6-atom hexagonal unit cell in $R\bar{3}m$ (166) symmetry with lattice parameters a = b = 4.63 Å and c = 12.25 Å, and the Bi atoms occupy the 6c (0, 0, 0.2674) Wyckoff position. The Bi(001) and Bi(012) surfaces are constructed by cleaving the bulk Bi structure, with a 15 Å vacuum layer along the Z direction, and are named as the Bi-001-i-Layer and Bi-012-i-Layer, respectively. The Bi-001-1-Layer is modeled by a periodic one-layer 4×4 slab. The lattice constants of the Bi-001-1-Layer are a = 18.54 Å, b = 18.54 Å, and c = 16.61 Å, respectively. The Bi-012-1-Layer is modeled by a periodic 1-layer 3×3 slab. The lattice constants of the Bi-012-1-Layer are a = 13.90 Å, b = 14.65 Å, and c = 15.23 Å. The structures of bismuthene are optimized with the convergence criteria for the electronic self-consistent relaxation and the ionic relaxation set to $10^{-6}$ eV and $10^{-2}$ eV/Å for energy and force, respectively. The Brillouin zone is sampled with a 3×3×1 Γ-centered Monkhorst–Pack k-point grid.

## 3. Results and Discussion

### 3.1. Surface Energy and Band Structures of Bismuthene

Bismuth (Bi), belonging to the space group $R\bar{3}m$ (166) and possessing time-reversal symmetry, is illustrated in the upper part of Figure 1a. It exhibits bulk–boundary correspondence under time-reversal symmetry protection. The unique physicochemical properties endow low-dimensional Bi metal catalysts with unparalleled catalytic performance compared to their bulk counterparts. The lower part of Figure 1a shows the Bi-001-9-Layer. The surface energy of few-layer bismuthene is calculated using the Equation (1) below:

(1) $$E_{\mathrm{surface}}=\frac{1}{2S}\left(E_{\mathrm{Bi-iL}}-n\cdot E_{\mathrm{Bi}}\right),$$

where $E_{\mathrm{surface}}$ represents the surface energy of the 2D material; $E_{\mathrm{Bi-iL}}$ denotes the energy of the *i*-layer bismuthene; $E_{\mathrm{Bi}}$ refers to the energy of a single atom in bulk Bi; *n* is the number of atoms in the unit cell of the *i*-layer bismuthene; and *S* is the surface area of the bismuthene. The surface energies of the Bi-001-1-layer, Bi-001-2-layer, Bi-001-3-layer, and Bi-001-9-layer bismuthene structures are 0.0088 eV·${Å}^{-2}$, 0.0098 eV·${Å}^{-2}$, 0.0102 eV·${Å}^{-2}$, and 0.0105 eV·${Å}^{-2}$, respectively. Surface energy analysis reveals that bismuthene structures with fewer layers exhibit lower surface energy and greater stability. As a result, monolayer bismuthene is more stable compared to multilayer structures. This computational result has also been experimentally validated, as monolayer Bi can be synthesized in the laboratory [45,46].

Band structure analysis shows a significant band overlap at the Fermi level in the band diagram of the Bi-001-1-Layer (Figure 1b), with the surface states indicated by red lines. The calculated results show that the valence and conduction bands of Bi-001-1-Layer intersect at the Γ-point on the Fermi level. As the number of Bi atomic layers increases, there is a slight increase in the number of bands near the Fermi surface, as shown in Figure 1c–e. As the film thickness increases (when the thickness is approximately above 34 Å), these bands will merge into a continuum in the bulk limit.

### 3.2. Free Energy Analysis of Intermediates in Bismuthene for ${\mathrm{CO}}_{2}$RR

DFT calculations were utilized to investigate the free energy of three reaction pathways on the Bi-001-1-Layer and Bi-012-1-Layer,

*OCO → *OCHO;
*OCO → *COOH;
*H → H_2_.

**Figure 2:**
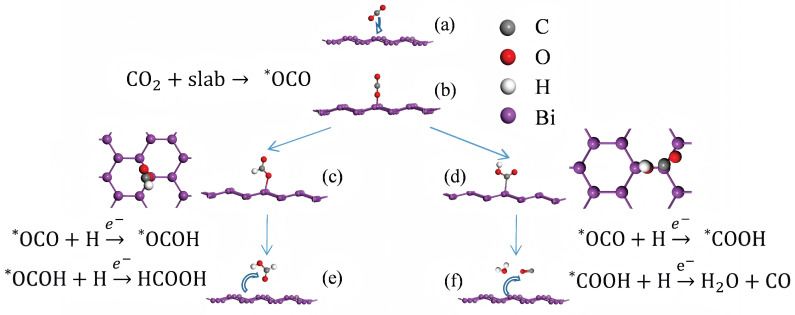
Degradation pathways of ${\mathrm{CO}}_{2}$ on the catalyst surface by first-principles calculations. (**a**) ${\mathrm{CO}}_{2}$ molecule adsorption, (**b**) *OCO adsorbed intermediate formation, (**c**) *OCOH adsorbed intermediate formation, (**d**) *COOH adsorbed intermediate formation, (**e**) formic acid molecule desorption from the catalyst surface, and (**f**) carbon monoxide and water molecule desorption from the catalyst surface.

Figure 2 presents a detailed illustration of the two potential reaction pathways for ${\mathrm{CO}}_{2}$RR. Based on thermodynamic data, the energy change for the formic acid production pathway in ${\mathrm{CO}}_{2}$RR was calculated to be 0.331 eV [47]. Since the complete reaction process involves two electron transfer steps, such as the formation of *COOH and formic acid, the average energy per step is 0.1655 eV.

**Figure 3:**
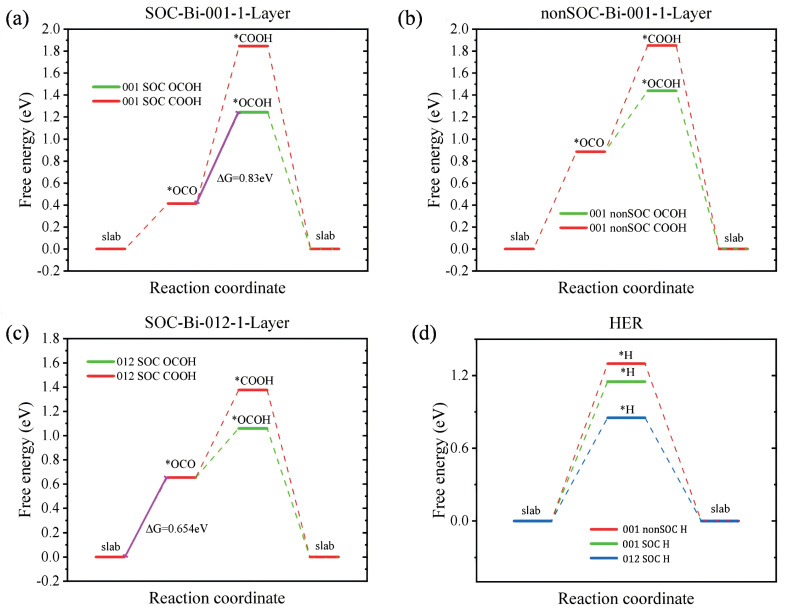
The Gibbs free energy change (ΔG) diagrams at U = 0.1655 V versus RHE are represented. (**a**,**b**) show the step diagrams of different ${\mathrm{CO}}_{2}$RR pathways on Bi-001-1-Layer with and without SOC effect, respectively. (**c**) The step diagram of different ${\mathrm{CO}}_{2}$RR pathways on Bi-012-1-Layer with SOC effect. (**d**) The step diagrams of HER on Bi-001-1-Layer with and without SOC effect, and the step diagrams of HER on Bi-012-1-Layer with SOC effect.

Table 1 lists the calculation results of reaction Gibbs free energies (G) of various intermediates in the ${\mathrm{CO}}_{2}$RR and the changes in Gibbs free energy (ΔG) at U = 0 V versus RHE and U = 0.1655 V versus RHE. The results of U = 0.1655 V versus RHE are represented in Figure 3. A comparison of Figure 3a,b reveals that calculations excluding SOC may overestimate the adsorption energy during the ${\mathrm{CO}}_{2}$RR process. The ΔG of the conversion of adsorbed ${\mathrm{CO}}_{2}$ molecules to *OCOH on Bi(001) and Bi(012) is 0.8303 eV and 0.654 eV, respectively. The ΔG of the conversion of adsorbed ${\mathrm{CO}}_{2}$ molecules to *COOH on Bi(001) and Bi(012) is 1.432 eV and 0.722 eV, respectively. It is observed that the free energy required for the conversion of adsorbed ${\mathrm{CO}}_{2}$ molecules to *COOH is greater than that for the conversion to *OCHO on both the Bi(001) and Bi(012) surface. This suggests that both the Bi(001) and Bi(012) active surfaces have high selectivity for the formation of *OCHO, the only catalytic product of the intermediate is formic acid, leading to the conclusion that the catalytic products of ${\mathrm{CO}}_{2}$RR on these substrates are formic acid. Moreover, bismuthene and Bi nanosheets have been experimentally confirmed to exhibit high electrocatalytic efficiency for formate (*OCOH) formation from ${\mathrm{CO}}_{2}$ reduction, with a Faradaic efficiency of 99% at −0.58 V vs. Reversible Hydrogen Electrode (RHE) for bismuthene and a Faradaic efficiency of 94.5% at −0.98 V vs. RHE for 1.5 nm Bi nanosheets [33,48].

Furthermore, the rate-determining step (RDS) of ${\mathrm{CO}}_{2}$RR on the (012) surface is the adsorption of *OCO with the ΔG of 0.654 eV (Figure 3c). And the RDS of (001) surface is the reaction of *OCO to *OCOH with the ΔG of 0.830 eV (Figure 3a). The lower RDS on (012) surface indicates that the Bi-012-1-Layer has higher catalytic efficiency than the Bi-001-1-Layer.

The ΔG free energies of *OCO, *OCOH, and *COOH on the Bi-001-1-Layer surface with SOC effect are 0.414 eV, 1.244 eV, and 1.846 eV and without SOC effect are 0.885 eV, 1.439 eV, and 1.851 eV, respectively, as shown in Table 1. These results show that the effect of SOC on the *COOH adsorption system is minimal, whereas its impact on *OCO and *OCOH are significant. As shown in Figure 2, *COOH adsorbs onto the catalyst surface via the C atom, whereas *OCO and *OCOH adsorb through the O atom. The O-Bi bond is highly sensitive to spin–orbit coupling. Bi, as a heavy element, exhibits a pronounced SOC effect, which is in accordance with our computational results.

Additionally, since ${\mathrm{CO}}_{2}$RR typically occurs in formate solutions, HER becomes the primary competing reaction on the catalyst surface. The reaction free energies (G) of HER on different exposed surfaces of the Bi-1-Layer (vibrationally corrected) are shown in Table 2. Considering the influence of SOC, the calculated adsorption free energies of *H on the Bi-001-1-Layer and Bi-012-1-Layer surfaces are 1.272 eV and 0.852 eV, respectively (see the green and blue dashed lines in Figure 3d). Both of them have larger energy barriers than the step of *OCO adsorption (0.414 and 0.654 eV) or the step of *OCO to *OCOH (0.830 and 0.590 eV) on the Bi-001-1-Layer and Bi-012-1-Layer surfaces, respectively. It is evident that ${\mathrm{CO}}_{2}$RR holds a greater reaction advantage over HER on these catalysts. Therefore, ${\mathrm{CO}}_{2}$RR is kinetically preferred over HER in the catalytic process.

### 3.3. Calculation and Analysis of Energy Band of Intermediates in Bismuthene for ${\mathrm{CO}}_{2}$RR

**Figure 4:**
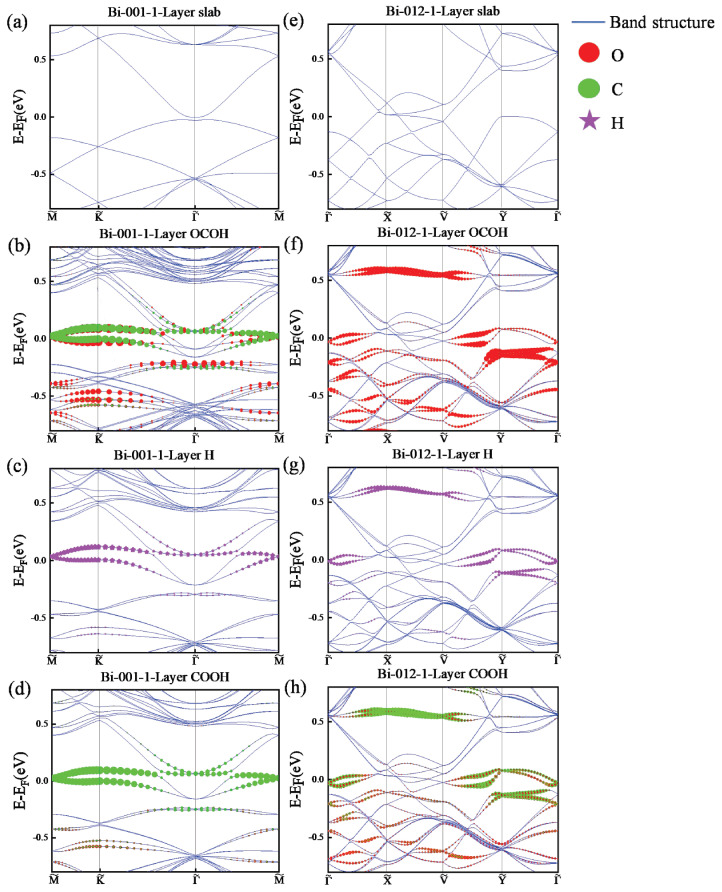
(**a**–**d**) The electronic band structures of the Bi(001) surface (**a**) without and with the adsorption of (**b**) *OCOH, (**c**) *H, and (**d**) *COOH intermediates with SOC effect, respectively. The high-symmetry points are $\tilde{M}$ (0.5, 0, 0), $\tilde{K}$ (0.333, 0.333, 0), and $\tilde{\Gamma }$ (0, 0, 0) in fractional coordinates. (**e**–**h**) The electronic band structures of the Bi(012) surface (**e**) without and with the adsorption of (**f**) *OCOH, (**g**) *H, and (**h**) *COOH intermediates with SOC effect, respectively. The high-symmetry points are $\tilde{\Gamma }$ (0, 0, 0), $\tilde{X}$ (0.5, 0, 0), $\tilde{V}$ (0.5, 0.5, 0), and $\tilde{Y}$ (0, 0.5, 0) in fractional coordinates. The red, green, and purple dots represent the projections of O, C, and H orbitals, respectively.

The electronic band structures of the Bi(001) surfaces without and with the adsorption of *OCOH, *H, and *COOH intermediates with SOC effect are plotted in Figure 4a–d, respectively. Our results show that there is a small gap in the electronic band structures of the one-layer Bi(001) surface at the $\tilde{\Gamma }$-point [see Figure 4a]. With the adsorption of *OCOH, *H, and *COOH intermediates, there are new states around the Fermi level, as shown in Figure 4b–d. From the projected electronic band structure of the Bi(001) surface with the adsorption of the *OCOH intermediate, we observe that the C and O orbitals contribute to the states near the Fermi level [see Figure 4b]. Similarly, for the Bi(001) surface with the adsorption of the *H intermediate, the H orbitals contribute to the states near the Fermi level [see Figure 4c]. From the projected electronic band structure of the Bi(001) surface with the adsorption of the *COOH intermediate, we find that the C orbitals contribute to the states near the Fermi level [see Figure 4d].

The electronic band structures of the Bi(012) surfaces without and with the adsorption of *OCOH, *H, and *COOH intermediates with SOC effect are also plotted in Figure 4e–h, respectively. The calculated results show that the one-layer Bi(012) surface is metallic [see Figure 4e]. From the projected electronic band structure of the Bi(012) surface with the adsorption of the *OCOH intermediate, we observe that the O orbitals contribute to the states near the Fermi level [see Figure 4f]. For the Bi(012) surface with the adsorption of the *H intermediate, the H orbitals make a minor contribution to the states near the Fermi level [see Figure 4g]. Also, for the Bi(012) surface with the adsorption of the *COOH intermediate, the C and O orbitals contribute to the states near the Fermi level [see Figure 4h]. Moreover, the adsorption of intermediates induces the splitting of electronic energy levels [see Figure 4]. These results indicate that different intermediates have distinct effects on the electronic properties of the systems. Specifically, on both the Bi(001) and Bi(012) surfaces, the projected electronic band structure of the *OCOH intermediate at the Fermi level is significantly more pronounced compared to that of the *H and *COOH intermediates. This indicates that both the intermediate and the system are more active when *OCOH is adsorbed, which could explain the higher catalytic activity associated with the adsorption of the *OCOH intermediate [33,48].

## 4. Conclusions

In conclusion, this work investigates the 2D bismuthene catalysts of the Bi(001) and Bi(012) surfaces in the ${\mathrm{CO}}_{2}$ reduction reaction through first-principles calculations. Surface energy calculations of monolayer and multilayer bismuthene confirmed that monolayer bismuthene is stable and suitable for catalytic applications on the Bi(001) surface. The adsorption free energy calculations suggest that both the Bi(001) and Bi(012) surfaces have high selectivity for the formation of *OCHO, leading to the fact that the catalytic products of ${\mathrm{CO}}_{2}$RR on these substrates are formic acid. Furthermore, the rate-determining step (RDS) of ${\mathrm{CO}}_{2}$RR on the (012) surface is the adsorption of *OCO with the ΔG of 0.654 eV. The RDS of the (001) surface is the reaction of *OCO to *OCOH with the ΔG of 0.830 eV. These results show the Bi(012) surface has a lower free-energy barrier than Bi(001) in the ${\mathrm{CO}}_{2}$RR process, representing higher catalytic activity. The calculated adsorption free energies of *H on the Bi-001-1-Layer and Bi-012-1-Layer surfaces are 1.272 eV and 0.852 eV, respectively. Both of them have larger energy barriers than the step of *OCO adsorption or the step of *OCO to *OCOH on the Bi-001-1-Layer and Bi-012-1-Layer surfaces, respectively. It is evident that ${\mathrm{CO}}_{2}$RR holds a greater reaction advantage over HER on these catalysts. By calculating the band structures of the Bi-001-1-Layer and Bi-012-1-Layer after the adsorption of various ${\mathrm{CO}}_{2}$RR intermediates, it is shown that the different intermediates have effects on the electronic properties of the systems. These results provide theoretical insights for designing bismuthene-based ${\mathrm{CO}}_{2}$RR catalysts with reduced overpotential (0.830 eV and 0.654 eV for Bi-001-1-layer and Bi-012-1-layer, respectively), enhanced selectivity, and improved electrocatalytic efficiency for formate (*OCOH) formation from ${\mathrm{CO}}_{2}$ reduction and also offer useful guidance for carbon dioxide reduction on other metal surfaces.

## Figures and Tables

**Table 1:** Reaction free energies (G) of various intermediates in the ${\mathrm{CO}}_{2}$RR on different exposed surfaces of Bi-1-Layer, as well as the changes in free energy at applied potentials of U = 0 V versus RHE and U = 0.1655 V versus RHE (vibrationally corrected).

Absorb Structure	G/eV	ΔG/eV (U = 0 V)	ΔG/eV (U = 0.1655 V)
Bi-SOC-001-1-Layer			
slab	−167.247	0.000	0.000
*OCO	−166.833	0.414	0.414
*OCOH	−165.837	1.410	1.244
*slab	−166.916	0.331	0.000
*COOH	−165.235	2.012	1.846
Bi-nonSOC-001-1-Layer			
slab	−148.332	0.000	0.000
*OCO	−147.447	0.885	0.885
*OCOH	−146.727	1.605	1.439
*slab	−148.001	0.331	0.000
*COOH	−146.359	1.973	1.851
Bi-SOC-012-1-Layer			
slab	−102.207	0.000	0.000
*OCO	−101.553	0.654	0.654
*OCOH	−100.983	1.224	1.059
*slab	−101.876	0.331	0.000
*COOH	−100.666	1.541	1.376

**Table 2:** Reaction free energies (G) of HER on different exposed surfaces of Bi-1-Layer (vibrationally corrected).

Absorb Structure	G/eV	ΔG/eV
Bi-SOC-001-1-Layer		
slab	−144.041	0.000
*H	−142.770	1.272
*slab	−144.041	0.000
Bi-nonSOC-001-1-Layer		
slab	−125.126	0.000
*H	−123.828	1.298
*slab	−125.126	0.000
Bi-SOC-012-1-Layer		
slab	−79.001	0.000
*H	−78.149	0.852
*slab	−79.001	0.000

## Data Availability

The original contributions presented in the study are included in the article, further inquiries can be directed to [ctwang@ustb.edu.cn].
